# Impact of peer review audit on occupational health report quality

**DOI:** 10.1093/occmed/kqv077

**Published:** 2015-06-04

**Authors:** D. Lalloo, E. Demou, E. B. Macdonald

**Affiliations:** ^1^NHS Lanarkshire, Salus Occupational Health, Safety & Return to Work Services, Hamilton ML3 0TA, UK,; ^2^Healthy Working Lives Group, Institute of Health and Wellbeing, College of Medical, Veterinary and Life Sciences, University of Glasgow, Glasgow G12 8RZ, UK,; ^3^MRC/CSO Social and Public Health Sciences Unit, Institute of Health and Wellbeing, College of Medical, Veterinary and Life Sciences, University of Glasgow, Glasgow G2 3QB, UK.

**Keywords:** Audit, occupational health, peer review, quality.

## Abstract

**Background:**

In a previous report, we described the implementation of a formal process for peer review of occupational health (OH) reports and a method of assessment of the outcomes of this process. The initial audit identified that 27% of OH reports required modifications.

**Aims:**

To assess formally, following implementation of this process, if changes in practice had occurred, i.e. whether fewer deficiencies were being identified in reports.

**Methods:**

We repeated a prospective internal audit of all peer reviewed OH reports between September and November 2011. We used an abbreviated assessment form, based on questions 4–8 and 10–12 of the modified SAIL (Sheffield Assessment Instrument for Letters), with four possible outcomes: no action, no changes made to report following discussion with author, changes made without discussion with author and changes made following discussion with author.

**Results:**

One hundred seventy-three reports by 10 clinicians were audited. The audit identified a 13% reduction in OH reports requiring modifications (from 27 to 14%) compared with the previous cycle. Where modifications were required, 8% of these were related to minor typographical, spelling and grammar errors and 6% were for more complex reasons. Implementation of this process also produced a reduction in clinical complaints about OH reports from customers, from three in the preceding year to none 2 years later.

**Conclusions:**

Peer review improved the standard of OH reports and was associated with a reduction in customer complaints about reports.

## Introduction

The role of peer review (i.e. structured evaluation of clinical work by colleagues in the same field) is expanding within clinical practice [1] as part of clinical governance and quality improvement [2,3] and with revalidation now established in the UK [4]. In a previous article [5], the importance of occupational health (OH) reports to managers and human resource professionals was highlighted, with issues relating to reports being identified as one of the commonest causes of complaints and customer dissatisfaction. A formal process for peer review of OH reports for selected customers was implemented and a method of assessing the outcomes of this process established. The initial audit identified that 27% of OH reports required modifications. Eighteen per cent of these related to minor errors, while in 9%, there were more complex reasons. Although the clinicians involved cited the process as a valuable educational tool and reported change in their practice, this had not been formally assessed. The purpose of this audit cycle was to assess formally, following implementation of a peer review process, whether changes in practice had in fact occurred, i.e. whether fewer deficiencies were being identified in reports, with a resulting improvement in the overall standard of reports.

## Methods

A prospective internal audit of all peer reviewed OH reports was repeated between September and November 2011. As previously, we used an abbreviated assessment form, based on questions 4–8 and 10–12 of the modified SAIL (Sheffield Assessment Instrument for Letters) [6,7]. Four key aspects of reports (administrative, response, professional issues and clarity) were graded on a 3-point rating scale (below expected, satisfactory and above expected) or were judged ‘not applicable’.

The peer review process detailed in the original art icle [5] was unchanged. One of four possible outcome options remained, i.e. no action, no changes made to report following discussion with author, changes made without discussion with author or changes made following discussion with author.

The completed forms were collated and analysed via the Survey Monkey™ online tool (SurveyMonkey.com, LLC, California, USA).

As this was a service audit involving the analysis of anonymized data from the outcome of the peer review process, ethical approval was not required. Management approval to undertake this work was obtained.

## Results

Eight reviewers (four consultant occupational physicians, one speciality registrar and three senior nurses) peer reviewed 173 reports by 10 clinicians over the 3-month audit period. The 10 clinicians whose reports were peer reviewed comprised the eight peer reviewers, one sessional doctor and an OH adviser. Each peer reviewer assessed a number of different clinicians’ reports and each clinician had reports assessed by a variety of reviewers. On this occasion, 14% of reports required modifications, compared with 27% previously. One year on, 86% of reports required no change, an increase from 71% in the previous year. The Fisher’s exact test demonstrated that the improvement in the standard of reports (Table 1) was statistically significant (*P* < 0.01). Where modifications were required, 8% of these were the result of minor errors and 6% for more complex reasons. Examples of issues identified are detailed in Table 1. The minor errors (typographical, spelling and grammar) were unchanged and remained the key reason for modifying reports. Among more complex reasons, reports not being clear and understandable to the intended readership and failure to address all the referrer’s questions remained areas for improvement. There was a substantial improvement in review arrangements being made clear in reports, with a 100% satisfactory score.

**Table 1.** Table of actions required following peer review, examples of changes September to November 2011 and comparison with first round

Action taken	Repeat cycle 2011, *n* (%)	First round 2010, *n* (%)	Examples of changes required 2011
No action—no changes to report required or no discussion with author required	148 (86)	152 (71)	
No changes made to report following discussion with author	0	4 (2)	Clarification only required, e.g. further background information required on the case or from previous correspondence
Changes made to report without discussion with author	14 (8)	40 (18)	Typographical, spelling or grammar errors
Changes made to report following discussion with author	11 (6)	19 (9)	Report not clear and understandable to the intended readership. All the referrer’s questions not addressed. OH advice not balanced
Total	173 (100)	215 (100)	

In addition to the audit process, internal consistency between the eight reviewers was formally assessed using 10 randomly selected reports that were separately assessed by each reviewer. Fleiss’s kappa statistic, a measure of agreement between three or more observers when the response is a group or category, was used for the calculation and the results are presented in Table 2. The majority of questions demonstrated fair or higher levels of agreement and only 2 out of the 11 had less than chance agreement between reviewers.

**Table 2.** Inter-observer agreement for all questions

Question	*κ*	Interpretation of *κ* [8]	Standard error	Lower CI	Upper CI
1. All fields completed correctly?	1	Perfect	0.06	0.88	1.12
2. Consent section fully complete?	1	Perfect	0.06	0.88	1.12
3. Is there a clear OH action plan or a specific set of recommendations?	0.39	Fair	0.05	0.29	0.49
4. Have all the manager’s questions been addressed? (directly, or in terms of the proposed plan)	0.74	Substantial	0.06	0.63	0.85
5. Are the OH review arrangements clear? (including decision not to offer follow-up)	0.85	Almost perfect	0.06	0.73	0.97
6. Where appropriate, are any legal and/or ethical issues highlighted? (e.g. Equality Act, consent)	0.45	Moderate	0.05	0.35	0.54
7. Adherence to contractual, ethical and legal boundaries?	0.02	Less than chance	0.06	−0.09	0.13
8. Is the OH advice balanced?	0.11	Slight	0.06	0.004	0.22
9. Is unnecessary information kept to a minimum?	0.03	Less than chance	0.06	−0.081	0.146
10.Does the structure of the report flow logically?	0.44	Moderate	0.06	0.33	0.56
11.Is the report clear and understandable to the intended readership?	0.16	Slight	0.01	0.15	0.17

CI, confidence interval.

## Discussion

The repeat audit identified a 13% reduction (from 27 to 14%) in OH reports requiring modifications. This may be related to numerous factors: a desire by the clinician to meet the standards, active feedback by the reviewers on specific areas for improvement and/or knowledge that the report would be reviewed by a peer.

This finding supports the evidence that medical audit feedback together with educational measures has some success in changing practice [9,10], particularly when delivered frequently and with specific suggestions for improvement [10].

The main strengths of this audit were the use of a validated assessment tool and the simplicity of the process. Internal consistency between reviewers was also addressed. This identified a good level of agreement for most questions and identified potential areas (e.g. adherence to contractual, ethical and legal boundaries) for reviewer training to improve our process further.

There were some concerns that peer review would breed complacency among clinicians, in the knowledge that their report would be ‘double checked’ and any deficiencies identified and actioned by the reviewer before being sent. We did not find any evidence to suggest this happens in practice. We intend to repeat the audit at intervals to assess if changes in practice are maintained. In time we hope that even fewer deficiencies will be identified and that peer review of samples, rather than of all reports, can be implemented.

Notably there were no complaints relating to the content of OH reports for these customers 2 years after the introduction of peer review compared with several the year before implementation. We will continue to monitor this. Customer feedback on OH reports since the process was formalized has also been positive although further work is required in terms of a formal survey.

We conclude that this peer review process not only improved the standard of OH reports but was also associated with a reduction in clinical complaints related to reports.

**Key points**

- In this study, peer review was a useful tool in improving the standard of occupational health reports.
- Its establishment has also led to a reduction in customer complaints.
- Inter-observer evaluation is an important factor in the interpretation of results and can highlight potential areas for reviewer training and process improvement.

## Funding

Medical Research Council (partnership grant MC/PC/13027 to E.D.).

## Conflicts of interest

None declared.
